# Frequent Missed Opportunities for Earlier HIV Diagnosis in a Routine Opt-out Testing Environment in Atlanta

**DOI:** 10.1093/ofid/ofaf423

**Published:** 2025-08-26

**Authors:** Sarah F Gruber, Megan Schwinne, Rishika Iytha, Emma J Hollenberg, Chad Robichaux, Valeria D Cantos, Jonathan A Colasanti, Anna Q Yaffee, Sara Turbow, Eric Leue, Andrés Camacho-González, Yun F Wang, Meredith H Lora

**Affiliations:** Department of Medicine, Emory University School of Medicine, Atlanta, Georgia, USA; Department of Biomedical Informatics, Emory University School of Medicine, Atlanta, Georgia, USA; Georgia Clinical and Translational Science Alliance, Emory University School of Medicine, Atlanta, Georgia, USA; Georgia Clinical and Translational Science Alliance, Emory University School of Medicine, Atlanta, Georgia, USA; Smith & Nephew, Pittsburgh, Pennsylvania, USA; Department of Medicine, Emory University School of Medicine, Atlanta, Georgia, USA; Department of Medicine, Hospital of the University of Pennsylvania, Philadelphia, Pennsylvania, USA; Department of Biomedical Informatics, Emory University School of Medicine, Atlanta, Georgia, USA; Georgia Clinical and Translational Science Alliance, Emory University School of Medicine, Atlanta, Georgia, USA; Division of Infectious Diseases, Department of Medicine, Emory University School of Medicine, Atlanta, Georgia, USA; Ponce de Leon Center, Grady Health System, Atlanta, Georgia, USA; Division of Infectious Diseases, Department of Medicine, Emory University School of Medicine, Atlanta, Georgia, USA; Ponce de Leon Center, Grady Health System, Atlanta, Georgia, USA; Department of Emergency Medicine, School of Medicine, Emory University, Atlanta, Georgia, USA; Grady Health System, Atlanta, Georgia, USA; Division of General Internal Medicine, Department of Medicine, Emory University School of Medicine, Atlanta, Georgia, USA; Division of Preventive Medicine, Department of Family and Preventive Medicine, Emory University School of Medicine, Atlanta, Georgia, USA; Ponce de Leon Center, Grady Health System, Atlanta, Georgia, USA; Grady Health System, Atlanta, Georgia, USA; Ponce de Leon Center, Grady Health System, Atlanta, Georgia, USA; Division of Infectious Diseases, Department of Pediatrics, Emory University School of Medicine, Atlanta, Georgia, USA; Children's Healthcare of Atlanta, Atlanta, Georgia, USA; Grady Health System, Atlanta, Georgia, USA; Department of Pathology and Laboratory Medicine, Emory University School of Medicine, Atlanta, Georgia, USA; Ponce de Leon Center, Grady Health System, Atlanta, Georgia, USA; Division of General Internal Medicine, Department of Medicine, Emory University School of Medicine, Atlanta, Georgia, USA

## Abstract

**Background:**

Routine, opt-out HIV testing strategies aim to diagnose HIV earlier and decrease ongoing transmission. We report missed opportunities (MO) and missed testing opportunities (MTO) for earlier HIV diagnosis in a safety-net healthcare system using routine opt-out testing.

**Methods:**

This retrospective study analyzed adults diagnosed with HIV between 2015 and 2022 with eligible encounters 30–365 days before diagnosis. MO is defined as no HIV testing in the year before diagnosis. MTOs are encounters where testing was indicated but not performed. Logistic regression identified factors associated with MO and MTO. To evaluate the opt-out testing program, we measured the number of individuals eligible for testing, tests completed, reactive results, and new HIV diagnoses.

**Results:**

Of 713 newly diagnosed individuals, 499 (70%) had MO. Among 1845 encounters, 1235 (67%) were MTO. Sexual health–related encounters and STI testing had lower MO odds (adjusted odds ratio [aOR] 0.62; 95% confidence interval [CI], 0.42–0.90; *P* = .013) and (aOR 0.36; 95% CI, 0.28–0.48; *P* < .0001), respectively. MO was associated with higher odds of CD4 < 350 cells/mm^3^ at diagnosis (aOR 1.8; 95% CI, 1.2–2.9; *P* = .011). Outpatient encounters, particularly primary care, had higher MTO odds than emergency department (OR 0.67; 95% CI, 0.54–0.82; *P* < .0001). Among 531,848 eligible individuals, 357,771 tests were conducted in 199,004 individuals (37.4%); 4719 (1.3%) tests were reactive, resulting in 861 (0.4%) new HIV diagnoses.

**Conclusions:**

Despite routine opt-out HIV testing, most individuals newly diagnosed with HIV had no testing in the prior year, highlighting the need to optimize opt-out testing procedures, particularly in primary care and nonsexual health visits.

## Teaser

Despite routine opt-out HIV testing, missed opportunities for earlier diagnosis remained prevalent among newly diagnosed individuals and were associated with advanced HIV. Optimized HIV testing, particularly in primary care and nonsexual health visits, is imperative to facilitate diagnosis and improve outcomes.

## BACKGROUND

In the United States, individuals with undiagnosed HIV account for 13% of all people with HIV (PWH) [1] but contribute to more than 40% of all new transmissions [2, 3], highlighting the need to improve HIV testing interventions.

Earlier HIV diagnosis via expanded testing is a primary pillar of the Ending the HIV Epidemic Initiative [2]. The US Centers for Disease Control and Prevention recommends 1-time opt-out HIV testing for all individuals aged 13–64 years without assessment of risk (i.e., routine opt-out HIV screening) and annual screening for persons with a higher likelihood of HIV acquisition [4–6]. Healthcare systems in the United States, including Grady Health System (GHS) in Atlanta, have adopted routine opt-out HIV screening. This method integrates HIV testing into standard care, offering it to patients by default unless they decline, unlike traditional opt-in testing, which requires patients to actively request the test.

Despite these efforts, recent studies have identified missed opportunities (MO) for earlier HIV testing among newly diagnosed PWH [2, 7–15]. Studies conducted in urban academic medical centers with similarities to GHS have demonstrated that persons perceived at a lower HIV risk are at higher risk for MO and that missed testing opportunities are most common in the emergency department (ED) [2, 8, 9 , 14]. Prior analyses of MO have been limited by small sample sizes [12] and varying definitions of MO that often do not consider testing indications [7–9, 11–14], and may be impacted by confounding factors related to healthcare access and overutilization [2, 7–11, 14].

We aimed to identify missed opportunities for earlier HIV diagnosis within our high-volume safety-net healthcare system in Atlanta, Georgia. Using a novel definition of missed opportunities that considers healthcare utilization and testing indications, we sought to quantify and characterize missed opportunities, identify associated risk factors, and evaluate the impact of MO on people diagnosed with HIV. To contextualize our findings, we analyzed the testing rate and diagnostic yield of our routine opt-out HIV screening program.

## METHODS

### Study Setting and Data Source

This retrospective cohort study was conducted at GHS, an urban safety-net hospital in Atlanta, Georgia. The center features a Level 1 trauma unit, handling 250,000 ED encounters and 120,000 primary care visits annually. It serves a catchment area of >6 million people living in Fulton and Dekalb counties [16], which are 2 of the 42 Ending the HIV Epidemic Initiative counties with the highest incidence of HIV in the United States [17].

In 2013, GHS implemented routine opt-out HIV testing in the ED and urgent care, and by 2017, this initiative was expanded to outpatient and inpatient settings. In the ED, an automatic electronic health record (EHR)-based best practice alert (BPA) was activated if an individual had not completed HIV testing in the prior 6 months. This alert prompted triage staff to offer HIV screening using normalizing language—emphasizing that testing is a routine and expected part of medical care to reduce potential discomfort or stigma—and to obtain verbal consent, in compliance with Georgia statute [18]. If the HIV test was not ordered, a second BPA was triggered when the provider accessed the individual's chart. In outpatient and inpatient settings, the BPA would activate if the last HIV screening occurred more than 1 year prior. Between 2020 and 2021, the GHS ED prioritized COVID-19–related BPAs, which resulted in the HIV screening BPA being moved from primary to secondary screening.

Data were extracted from the GHS Epic EHR database. Extracted data included patient demographics, encounter details, laboratory tests and results, and diagnosis codes.

### Patient Consent Statement

This research was approved by the Emory University institutional review board and the GHS Research Oversight Committee with a waiver for informed consent. No patient images or otherwise patient-identifying information are included in this manuscript.

### Study Population

**Figure 1.**
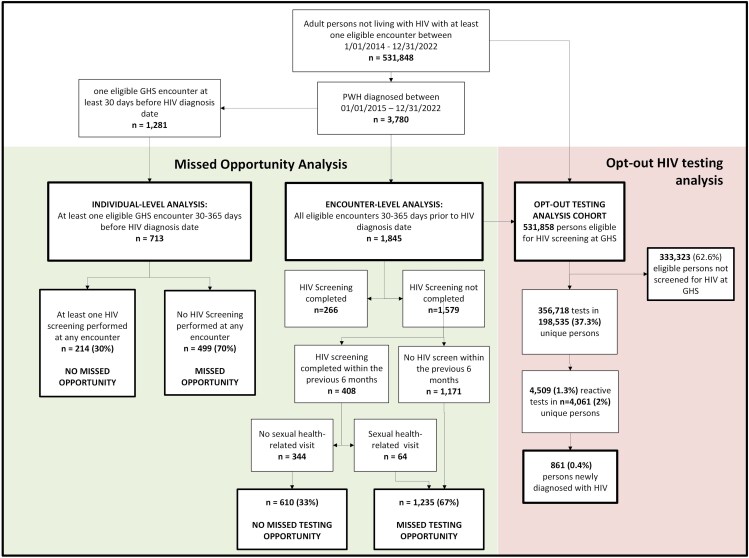
Cohort definitions for individual-level and encounter-level analyses and analysis of opt-out testing system. Abbreviations: ED, emergency department; GDPH, Georgia Department of Public Health; GHS, Grady Health System; IP, inpatient; OP, outpatient; UC, urgent care.

All adult individuals with at least 1 encounter at GHS between 01 January 2014 and 31 December 2022 were included. PWH were defined as anyone in the cohort with a diagnosis of HIV per the Georgia Department of Public Health [19]. Eligible testing encounters were defined as provider-based encounters within departments where opt-out testing was implemented (e.g., inpatient, emergency, urgent care, selected outpatient settings, including primary care, women's health, dermatology, medical subspecialty clinics). Visits for nursing encounters, procedures, imaging, therapy, and surgical specialties were excluded since opt-out testing was not implemented in these settings. PWH with an HIV diagnosis date between 2015 and 2022 and at least 1 eligible GHS healthcare encounter 30–365 days before their diagnosis were included in the analysis for missed opportunities (Figure 1). Encounters within 30 days of the HIV diagnosis date were excluded to avoid miscategorizing the GHS encounter where HIV diagnosis occurred as an MO. To prevent overcounting of linked encounters (e.g., an ED encounter that led to an admission), encounters within 3 days of each other were named “encounter groups” and analyzed as a single encounter.

To evaluate our opt-out testing program, we included all people eligible for HIV testing at GHS, defined as persons not living with HIV before 2014 who had at least 1 eligible testing encounter during the study period.

### Primary Outcome: Missed Opportunity at the Individual Level

The primary study outcome was MO, defined as people who did not receive HIV testing at any eligible encounter group 30–365 days before their HIV diagnosis date.

### Secondary Outcome: Missed Testing Opportunity at the Encounter Level

An encounter was considered a missed opportunity for HIV testing (MTO) if it occurred 30–365 before an HIV diagnosis and HIV testing was indicated but not performed. Testing was considered indicated if at least 1 of the following conditions were met: the encounter (1) occurred more than 6 months after the last HIV test or (2) included a documented sexual health–related diagnosis or chief complaint. These conditions were chosen based on GHS BPA firing parameters (criteria 1) and US Centers for Disease Control and Prevention testing guidelines for testing and prior studies (criteria 2) [14, 20].

### Variable Selection

We cleaned the extracted EHR data using R version 4 statistical software [21] and created variables encompassing sociodemographic, healthcare utilization, and clinical information. The selection of variables was guided by an extensive literature review and collaboration between clinicians and biostatisticians to ensure their relevance. A complete list of variables and the data dictionary can be found in the Supplementary material. We generated a correlation heatmap to address multicollinearity, identifying pairs of variables with high correlation values. Only those variables with sufficient data and without multicollinearity were retained for further analysis.

### Statistical Analysis—Primary Outcome, MO at the Individual Level

All statistical analyses were performed in R version 4 statistical software [22]. Descriptive statistics were computed for the overall study population and stratified by the presence of MO. Categorical variables were summarized using frequencies and column percentages, wheres continuous variables were expressed as means with standard deviations. Using univariable and multivariable logistic regression, we estimated odds ratios (OR), 95% confidence intervals (CI), and *P* values for the association between each descriptive variable and MO. Statistical significance was set at *P* < .05 for all analyses. Age, race, sex, number of encounters in the 30–365 days before diagnosis, and HIV testing in the 2 years before diagnosis were identified as potential confounders and controlled for in the multivariable analysis.

### Statistical Analysis—Secondary Outcome, Encounter-level MTO

We stratified all relevant encounters by the presence of an MTO. Univariable logistic regression was again employed to analyze the association between encounter-level descriptive variables and MTO (secondary outcome), presented as OR (95% CI). We used Firth's logistic regression for more reliable parameter estimation to address potential bias caused by predictors that perfectly separated groups (e.g., when 1 group had either 0% or 100% of the outcome). Statistical significance was set at *P* < .05 for all analyses.

### Analysis of Routine Opt-out HIV Testing Program

In a secondary analysis, we identified the number of individuals who were eligible for HIV testing and how many of those received HIV testing at GHS during our study period. To determine the prevalence of positive test results and the number of people newly diagnosed with HIV, we identified all accurate positive tests and individuals with at least 1 positive HIV test. A positive HIV test was defined as a positive Architect HIV Antigen/Antibody Combination Assay. New diagnoses of HIV were defined as individuals with positive HIV testing at GHS occurring within 30 days of the Georgia Department of Public Health diagnosis date.

## RESULTS

### Primary Outcome—Individual-level Missed Opportunities (MO)

Among PWH diagnosed during the study period per the GDPH database, 713 individuals had at least 1 eligible encounter at GHS in the 30–365 days before diagnosis and were included for analysis of missed opportunities. Of the 713 individuals, 54% were aged 20–34 years (median 32 years; interquartile range [IQR] 24 years), 66% (471/713) were male, and 91% (629/713) were Black. Of these individuals, 70% (499/713) met the MO definition (i.e., they had no HIV testing at any encounter in the year preceding HIV diagnosis, which consisted of 1098 encounters, including 501 outpatient, 551 ED/urgent care, and 46 inpatient encounters). Individuals with MO had a median of 1.0 encounters (IQR 1–3) the year before diagnosis (Table 1).

**Table 1.** Characteristics of Individuals Diagnosed With HIV in Health System 2015–2022

Demographics, n (%)	Cohort (n = 713)	Missed Opportunity^a^		Univariable		Multivariable	
		No (n = 214, 30%)	Yes (n = 499, 70%)	OR (95% CI)	*P* Value	OR (95% CI)	*P* Value
Age at diagnosis, y							
20–34	383 (54%)	142 (66%)	241 (48%)	Ref		Ref	
35–49	156 (22%)	41 (19%)	115 (23%)	1.7 (1.1–2.5)	.017	2.1 (1.3–3.4)	.002
50+	174 (24%)	31 (15%)	143 (29%)	2.7 (1.8–4.3)	<.0001	3.4 (2.2, 5.7)	<.0001
Legal sex							
Male	471 (66%)	146 (68%)	325 (65%)	Ref		Ref	…
Female	242 (34%)	68 (32%)	174 (35%)	1.1 (0.82–1.6)	.43	1.4 (0.93–2.0)	.12
Race^b^							
White	45 (6.5%)	11 (5.1%)	34 (6.8%)	Ref		Ref	
Black	629 (91%)	189 (88%)	440 (88%)	0.75 (0.36–1.5)	.43	0.92 (0.42–1.9)	.83
Other	14 (2.0%)	6 (2.8%)	8 (1.6%)	0.43 (0.12–1.6)	.19	0.75 (0.19–3.0)	.68
Ethnicity^b^							
Hispanic	25 (3.5%)	8 (3.7%)	17 (3.4%)	Ref		Ref	
Non-Hispanic	677 (95%)	201 (94%)	476 (95%)	1.1 (0.45–2.5)	.80	1.2 (0.20–6.0)	.85
TGW^b^	12 (1.7%)	6 (2.8%)	6 (1.2%)	0.42 (0.13–1.4)	.14	0.99 (0.28–3.6)	.99
MSM^b^	51 (7.2%)	17 (7.9%)	34 (6.8%)	0.85 (0.47–1.6)	.59	1.2 (0.60–2.4)	.66
Black MSM^b^	40 (5.6%)	12 (5.6%)	28 (5.6%)	1.0 (0.51–2.1)	.99	1.3 (0.63–2.8)	.47
Black Female^b^	216 (30%)	61 (29%)	155 (31%)	1.1 (0.80–1.6)	.50	0.85 (0.21–3.2)	.89

Abbreviations: CI, confidence interval; MSM, men who have sex with men; OR, odds ratio; TGW, transgender woman.

^a^In this context, a missed opportunity is defined as an individual who was not screened for HIV in all relevant encounters 30–365 days before HIV diagnosis or screened 30 days prior. This outcome is stratified by those with all missed opportunities in the year prior to diagnosis vs. those who had no or some missed opportunities for HIV screening.

^b^Data are incomplete due to missing data.

Individuals aged 35–49 years and older than 50 years had higher odds of MO compared to those aged 20–34 (adjusted OR [aOR] 2.1 [95% CI, 1.3–3.4] and aOR 3.4 [2.2–5.7], respectively, *P* < .0001). There was a trend toward women having higher odds of MO than men (aOR 1.4 [0.93–2.0], *P* = .12). There were no associations between race, ethnicity, gender identity, or sexual orientation and the odds of MO (Table 1).

For individuals with MO, the median number of days between HIV diagnosis date and last negative HIV screening was 715 days (IQR 634), compared to 194 days (IQR 160) for those without MO. Individuals who received screening in the 1–2 years before HIV diagnosis had higher odds of receiving testing the following year (aOR for MO 0.41 [0.27–0.63], *P* < .0001) (Table 2). Individuals with MO had more than double the odds of having CD4 < 350 cells/mm^3^ at diagnosis (aOR 1.8 [1.2–2.9], *P* = .011).

**Table 2.** Factors Predictive of Having a Missed Opportunity for HIV Screening Among Individuals Diagnosed With HIV 2012–2022^a^

n (%)	Missed Opportunity^b^		Univariable		Multivariable	
	No (n = 214, 30%)	Yes (n = 499, 70%)	OR (95% CI)	*P* value	Adjusted OR (95% CI)	*P* value
HIV-related factors						
HIV screened 366–730 days Before Dx	65 (30%)	68 (14%)	0.36 (0.25–0.53)	<.0001	0.41 (0.27–0.63)	<.0001
CD4 < 200	13 (6.1%)	63 (13%)	2.2 (1.2–4.2)	.014	1.7 (0.92–3.4)	.11
CD4 < 350	29 (14%)	133 (27%)	2.3 (1.5–3.6)	.0002	1.8 (1.2–2.9)	.011
Healthcare utilization						
No. of encounters 30–365 days before Dx^c^	3.5 (3.5)	2.2 (2.3)	0.48 (0.30–0.75)	<.0001	0.83 (0.77–0.89)	<.0001
No. of encounters ever^a^	92 (146)	76 (121)	1.0 (0.99–1.0)	.13	1.0 (0.99–1.0)	.027
>2 visits to ED/UC 2 y ≤ Dx	103 (48%)	142 (28%)	0.43 (0.31–0.60)	<.0001	0.68 (0.46, 1.0)	.054
ED encounter without labs 2y ≤ Dx	113 (53%)	332 (67%)	1.8 (1.3–2.5)	.0007	2.6 (1.8–3.7)	<.0001
STI exposure complaint 2 y ≤ Dx	25 (12%)	15 (3.0%)	0.23 (0.12–0.45)	<.0001	0.43 (0.21–0.88)	.022
SH-related complaint 2 y ≤ Dx	99 (46%)	138 (28%)	0.44 (0.32–0.62)	<.0001	0.62 (0.42–0.90)	.013
No SH-related visit	107 (50%)	332 (67%)	2.0 (1.4–2.8)	<.0001	1.4 (0.98–2.1)	.064
Behavioral/mental health						
STI 2 y ≤ Dx	35 (16%)	26 (5.2%)	0.28 (0.16–0.48)	<.0001	0.47 (0.26–0.83)	.009

Abbreviations: CI, confidence interval; ED, emergency department; IV, intravenous; Meth, methamphetamine; OR, odds ratio; PC, primary care; PID, pelvic inflammatory disease; SDOH, social determinants of health; SH, sexual health; STI, sexually transmitted infection; UC, urgent care; WH, women's health.

^a^This table has been condensed to display only factors which reached statistical significance. A full report of factors evaluated can be found in Supplementary Table 1.

^b^In this context, a missed opportunity is defined as an individual who was not screened for HIV in all relevant encounters 30–365 days before HIV diagnosis or screened 30 days prior. This outcome is stratified by those with all missed opportunities in the year prior to diagnosis vs. those who had no or some missed opportunities for HIV screening.

^c^Represented as μ (σ).

Several factors related to healthcare utilization were significantly associated with the odds of MO. Individuals without MO had more healthcare visits in the year before diagnosis compared to those with MO (3.5 encounters versus 2.3; aOR 0.83 [0.76–0.89], *P* < .0001). In univariate analysis, individuals with at least 2 encounters in the ED or urgent care in the 2 years before diagnosis had lower odds of MO (OR 0.43 [0.31–0.60], *P* < .0001). This finding was maintained with multivariate analysis, although the association only trended toward statistical significance (aOR 0.68 [0.46–1.0], *P* = .054). In contrast, individuals with at least 1 ED encounter without a blood draw for other testing in the 2 years before HIV diagnosis had nearly double the odds of MO (aOR 2.6 [1.8–3.7], *P* < .0001). The number of primary care encounters in the 2 years before HIV diagnosis was similar between individuals with and without MO (aOR 1.0 [0.99–1.0], *P* = .25) (Table 2).

Individuals who sought care for sexually transmitted infection (STI) exposure or other sexual health–related complaints in the 2 years before diagnosis had lower odds of MO (aOR 0.43 [0.21–0.88], *P* = .022 and aOR 0.62 [0.42–0.90], *P* = .013, respectively). Similarly, individuals without a sexual health–related encounter had double the odds of MO in unadjusted models (OR 2.0 [1.4–2.8], *P* < .0001), although the statistical significance of this association was not maintained after controlling for confounding variables (aOR 1.4 [0.98–2.1], *P* = .064). Social determinants of health (SDOH) were not found to be associated with MO (Table 2).

### Secondary Outcome—Encounter Level Missed Testing Opportunities

Of the 1845 testing-indicated encounters that occurred 30–365 days before an HIV diagnosis, 1235 (67%) encounters were MTO (Table 3). Within clinical settings, with outpatient as the reference group, MTO were less common in the ED/urgent care (OR 0.67 [0.54–0.82], *P* < .0001). Within outpatient departments, compared to primary care, women's health departments (i.e., obstetrics and gynecology) had lower odds of MTO (OR 0.37 [0.24–0.57], *P* < .0001). Encounters with any bacterial STI testing had lower odds of being an MTO (OR 0.36 [0.28–0.48], *P* < .0001), and this was true for each bacterial STI independently (gonorrhea: OR 0.33 [0.28–0.48]; chlamydia: OR 0.34 [0.25–0.46]; and syphilis: OR 0.25 [0.17–0.36]; *P* < .0001). The presence of SDOH, mental health, and substance use diagnoses were not associated with the odds of MTO.

**Table 3.** Encounter Characteristics:^a^ Labs, Diagnoses, and Encounter Locations on Relevant Encounters^b^ in the Year Prior to HIV Diagnosis

n (%)	Encounters (n = 1845)	No Missed HIV Testing Opportunity (n = 610; 33%)	Missed HIV Testing Opportunity^c^ (n = 1235; 67%)	Odds Ratio (95% CI)	*P* Value
Encounter types					
Outpatient	762 (41%)	213 (35%)	549 (44%)	Ref	
ED/UC	1003 (54%)	369 (60%)	634 (51%)	0.67 (0.54–0.82)	<.0001
Inpatient	80 (4.3%)	28 (4.6%)	52 (4.2%)	0.72 (0.45–1.2)	.19
Outpatient departments^d^					
PC	470 (62%)	116 (55%)	354 (65%)	Ref	
OB/GYN	109 (14%)	51 (24%)	58 (11%)	0.37 (0.24–0.57)	<.0001
Specialty	183 (24%)	46 (22%)	137 (25%)	0.98 (0.66–1.5)	.90
Lab tests taken					
Bacterial STI	257 (14%)	138 (23%)	119 (9.6%)	0.36 (0.28–0.48)	<.0001
Gonorrhea	193 (11%)	110 (18%)	83 (6.7%)	0.33 (0.24–0.44)	<.0001
Syphilis	123 (6.7%)	79 (13%)	44 (3.6%)	0.25 (0.17–0.36)	<.0001
Chlamydia	196 (11%)	110 (18%)	86 (7.0%)	0.34 (0.25–0.46)	<.0001
Behavioral/sexual health					
STD exposure complaint	36 (2.0%)	18 (3.0%)	18 (1.5%)	0.49 (0.25–0.94)	.045
SDOH Dx	86 (4.7%)	37 (6.1%)	49 (4.0%)	0.64 (0.41–0.99)	.058

Abbreviations: Dx, diagnosis; ED, emergency department; Meth, methamphetamines; OB/GYN, obstetrician and gynecologist; PC, primary care; SDOH, social determinants of health; SH, sexual health; Specialty, specialty clinics (e.g., dermatology; cardiology); STD, sexually transmitted disease; STI, sexually transmitted disease (encompasses gonorrhea, syphilis, or chlamydia); UC, urgent care.

^a^This table has been condensed to display only encounter characteristics that reached statistical significance. A full report of encounter characteristics analyzed can be found in Supplementary Table 2.

^b^Relevant encounters are 30–365 days before HIV diagnosis that were in primary care, ED, UC, or inpatient.

^c^In this context, a missed HIV testing opportunity is defined as an encounter 30–365 days before HIV diagnosis in which they were not screened within 6 months or not screened upon an encounter in which there was a sexual health related chief complaint or diagnosis.

^d^PC, OB/GYN, and Specialty are departments from outpatient encounters. Therefore, their column % only considers outpatient encounters.

### GHS Routine Opt-out HIV Testing Program Analysis

Between 2014 and 2022, 531,848 individuals were eligible for opt-out routine HIV screening at GHS. 357,771 tests were performed on 199,004 unique individuals (37.4%). Of these, 4719 tests (1.3% of tests) were reactive, producing positive results in 4211 individuals (2.1% of individuals) and resulting in 861 (0.4% of individuals) new diagnoses of HIV (Figure 1).

## DISCUSSION

In this 7-year retrospective study, we characterized MO for HIV testing among individuals newly diagnosed with HIV in a large, safety-net hospital system using a routine opt-out HIV testing approach. In our study population, 70% of individuals newly diagnosed with HIV were not tested for HIV at any encounter in our health system in the year preceding diagnosis. This proportion of MO is double what has been previously reported [2, 9]. Factors associated with higher odds of MO included being older than 35 years of age, lower healthcare utilization, ED visits without blood drawn for other testing, and visits unrelated to sexual health. Outpatient encounters, particularly in primary care, were more likely to represent MTO than ED visits. Notably, individuals with MO experienced longer periods of undiagnosed HIV and, in contrast to prior studies, had nearly twice the odds of presenting with advanced HIV, defined as a CD4 count less than 350 cells/mm^3^ at diagnosis, compared to those without MO [2, 9]. Delayed diagnosis and advanced disease at presentation are associated with more significant morbidity and mortality for PWH [22–25]. Further, a recent modeling study showed that individuals with chronic HIV infection who were unaware of their status had more than double the transmission rate compared to PWH in care, regardless of viral suppression [3]. These individual and public health impacts underscore the urgent need to address missed opportunities to facilitate earlier linkage to HIV care. We analyzed our opt-out testing program to contextualize our results and found that despite an adequate overall positive test prevalence of 1.3% [11, 26], only about one third of all eligible individuals were tested for HIV. These results, particularly in the context of clinically significant missed opportunities, highlight the need to optimize our routine opt-out HIV testing strategy.

Contrary to prior studies, we found that outpatient encounters, particularly in primary care, were more likely to be MTO [2, 15]. Although primary care providers are well positioned to incorporate HIV testing into routine care due to their long-term relationships with patients [27], they face significant barriers. These include persistent HIV-related stigma, particularly in Black communities in the Southern United States [28], and associated concerns about straining the physician-patient relationship by offering testing [27]. Furthermore, primary care providers are uniquely vulnerable to systemic limitations, including time-constrained visits and competing priorities [29–31], with a simulation study demonstrating they would require 26.7 hours daily to deliver all recommended care [31]. Digital strategies for direct-to-patient education outside of clinical visits could help reframe HIV testing as routine, reduce HIV-related stigma, encourage patient-initiated testing, and save valuable time during encounters [32–34]. Further studies examining MO in the primary care setting are needed.

Unsurprisingly, seeking sexual health–related care was protective against MO and MTO. In line with prior studies [35, 36], we hypothesize that this finding reflects HIV testing based on risk perception by clinicians. However, nearly half of newly diagnosed PWH did not seek sexual health-related care during the study period, and lack of sexual health–related care nearly doubled the odds of MO, indicating that this risk perception is inaccurate and/or incomplete. While testing based on inaccurate risk perception is problematic, accurate risk assessment that reflects epidemiologic and behavioral factors could optimize HIV testing [37]. Emerging studies have developed HIV risk scores using machine learning algorithms [11, 38–40]. A hybrid strategy combining routine testing with validated HIV risk assessment tools may enhance screening, support early diagnosis, and broaden prevention through provider- or patient-directed prompts.

Our study has several limitations. First, this is a single-site study, so our results may not be generalizable. Second, we were unable to determine why HIV testing did not occur during specific encounters, whether because of the provider's oversight, patient decision to opt out, or other factors. Additionally, PWH in our database that did not seek care at GHS the year before diagnosis were excluded from our study. There is a possibility that some of these individuals were tested for HIV outside of our system, but we were unable to capture it. Although we did not find an association between race and MO, bias may still play a role in other settings and warrants further investigation. Finally, individuals with at least 1 HIV test in the year before diagnosis were categorized as not having MO, which could have led to an underestimation of missed opportunities in cases where more frequent HIV testing was clinically indicated.

This study provides a comprehensive evaluation of a real-world HIV opt-out testing strategy within 1 of the largest safety-net healthcare systems in the Southern United States, serving a population with 1 of the highest HIV incidence rates in the nation. In a novel approach, we used a statewide HIV diagnosis database to identify newly diagnosed PWH and combined these data with our internal database, enabling us to include a broader study population who had interacted with our healthcare system before their HIV diagnosis. Additionally, we employed a more specific definition of MO than previous studies [2, 8, 9, 13, 14], requiring no HIV testing during any encounter in the year before diagnosis and reducing the likelihood that overutilization of healthcare confounds our findings. These innovations allowed us to quantify the prevalence of clinically and temporally significant missed opportunities and offer clinical and public health insight into the impact of MO on efforts to end the HIV epidemic. Our findings prompt further exploration of the barriers and facilitators of our opt-out testing program and to develop, implement, and study interventions to address them.

## CONCLUSIONS

Despite a systemwide opt-out HIV testing program, most individuals newly diagnosed with HIV in a high-volume safety-net healthcare system had missed opportunities for earlier HIV diagnosis. These missed opportunities have important clinical and public health implications and underscore the need to optimize HIV testing strategies to facilitate earlier HIV diagnosis.

## Supplementary Material

ofaf423_Supplementary_Data

## Supplementary Data

Supplementary materials are available at *Open Forum Infectious Diseases* online. Consisting of data provided by the authors to benefit the reader, the posted materials are not copyedited and are the sole responsibility of the authors, so questions or comments should be addressed to the corresponding author.

## References

[1] Burns DN, DeGruttola V, Pilcher CD, et al Toward an endgame: finding and engaging people unaware of their HIV-1 infection in treatment and prevention. AIDS Res Hum Retroviruses 2014; 30:217–24. 24410300 10.1089/aid.2013.0274 PMC3938938

[2] Paer J, Ratcliffe J, Chang M, et al Predictors of missed HIV screening opportunities among newly diagnosed individuals at an urban medical center in New York City. PLoS One 2023; 18:e0290414. 37676864 10.1371/journal.pone.0290414 PMC10484428

[3] Baxter A, Gopalappa C, Islam MH, et al Updates to HIV transmission rate estimates along the HIV care continuum in the United States, 2019. J Acquir Immune Defic Syndr 2025; 99:47–54. 10.1097/QAI.0000000000003623 PMC11981839 39847445

[4] Branson BM, Handsfield HH, Lampe MA, et al Revised recommendations for HIV testing of adults, adolescents, and pregnant women in health-care settings. MMWR Recomm Rep 2006; 55(RR-14):1–17. 16988643

[5] Soh Q, Oh L, Chow E, Johnson C, Jamil M, Ong J. HIV testing uptake according to opt-in, opt-out or risk-based testing approaches: a systematic review and meta-analysis. Curr HIV/AIDS Rep 2022; 19:375–83. 10.1007/s11904-022-00614-0 PMC9508204 35829949

[6] Traynor SM, Rosen-Metsch L, Feaster DJ. Missed opportunities for HIV testing among STD clinic patients. J Community Health 2018; 43:1128–36. 10.1007/s10900-018-0531-z PMC6212336 29796786

[7] Guess S, Gormley MA, Moschella P, Roth P, Litwin AH. Missed opportunities for diagnosis of HIV in the emergency department using non-risk-based testing strategy. J Am Coll Emerg Physicians Open 2023; 4:e12898. 10.1002/emp2.12898 PMC9930739 36817078

[8] DeRose J, Zucker J, Cennimo D, Swaminathan S. Missed testing opportunities for HIV screening and early diagnosis in an urban tertiary care center. AIDS Res Treat 2017; 2017:1–6. 10.1155/2017/5708620 PMC5514340 28744377

[9] Zucker J, Patterson B, Ellman T, et al Missed opportunities for engagement in the prevention continuum in a predominantly Black and Latino community in New York City. AIDS Patient Care STDS 2018; 32:5. 10.1089/apc.2018.0127 PMC6247377 30398951

[10] Lyons MS, Lindsell CJ, Wayne DB, et al Comparison of missed opportunities for earlier HIV diagnosis in 3 geographically proximate emergency departments. Ann Emerg Med 2011; 58:S12–S21. 10.1016/j.annemergmed.2011.03.018 PMC3690121 21684399

[11] Weissman S, Yang X, Zhang J, Chen S, Olatosi B, Li X. Using a machine learning approach to explore predictors of health care visits as missed opportunities for HIV diagnosis. AIDS 2021; 35:S7–S18. 10.1097/QAD.0000000000002735 PMC8172090 33867485

[12] Chin T, Hicks C, Samsa G, McKellar M. Diagnosing HIV infection in primary care settings: missed opportunities. AIDS Patient Care STDS 2013; 27:392–7. 10.1089/apc.2013.0099 PMC3704080 23802143

[13] Downing A, Garcia-Diaz JB. Missed opportunities for HIV diagnosis. J Int Assoc Provid AIDS Care 2017; 16:15–7. 10.1177/2325957416661423 27496867

[14] Anderson S, Friedman EE, Eller D, et al HIV testing in a high prevalence urban area in the US: identifying missed opportunities two ways. Int J STD AIDS 2022; 33:970–7. 10.1177/09564624221118484 PMC10311933 36031933

[15] Powell M, Krentz HB, Eagles ME, Gill MJ. Missed opportunities within healthcare for an earlier diagnosis of HIV. Int J STD AIDS 2020; 31:1169–71. 10.1177/0956462420945948 32936718

[16] Atlanta metro area now 6th largest in the U.S . Metro Atlanta Chamber. **2024**. Available at: https://www.metroatlantachamber.com/atlanta-metro-area-now-6th-largest-in-the-u-s/. Accessed 16 December 2024.

[17] Piske M, Nosyk B, Smith J, et al Ending the HIV epidemic in metropolitan Atlanta: a mixed-methods study to support the local HIV/AIDS response. J Int AIDS Soc 2024; 27:e26322. 10.1002/jia2.26322 PMC11263453 39039716

[18] Georgia General Assembly . Georgia Code § 31-22-9.2 – HIV Tests: Notification, Counseling, and Exceptions. Atlanta, GA: State of Georgia, 2024. https://law.justia.com/codes/georgia/title-31/chapter-22/section-31-22-9-2/. Accessed 1 February 2025.

[19] Centers for Disease Control and Prevention . Technical Guidance for HIV Surveillance Programs: Adult HIV Confidential Case Report Form (National HIV Surveillance System Technical Guidance—ACRF). Atlanta, GA: Centers for Disease Control and Prevention, 2023. Available at: https://dph.georgia.gov/document/document/hivepiuser-guideadult-case-report-form2023pdf/download. Accessed 27 January 2025.

[20] CDC . HIV Nexus: CDC resources for Clinicians. Clinical Testing Guidance for HIV. Atlanta, GA: U.S. Department of Health & Human Services, 2024. Available at: https://www.cdc.gov/hivnexus/hcp/diagnosis-testing/index.html. Accessed 14 September 2024.

[21] R Core Team . R: a language and environment for statistical computing. Vienna, Austria: R Foundation for Statistical Computing, 2025. Available at: http://rstudio.com. Accessed 27 January 2025.

[22] Hecht FM, Wang L, Collier A, et al A multicenter observational study of the potential benefits of initiating combination antiretroviral therapy during acute HIV infection. J Infect Dis 2006; 194:725–33. 16941337 10.1086/506616

[23] Smith MK, Rutstein SE, Powers KA, et al The detection and management of early HIV infection: a clinical and public health emergency. J Acquir Immune Defic Syndr 2013; 63:S187–S199. 23764635 10.1097/QAI.0b013e31829871e0 PMC4015137

[24] Strain MC, Little SJ, Daar ES, et al Effect of treatment, during primary infection, on establishment and clearance of cellular reservoirs of HIV-1. J Infect Dis 2005; 191:1410–8. 10.1086/428777 15809898

[25] Ananworanich J, Dubé K, Chomont N. How does the timing of antiretroviral therapy initiation in acute infection affect HIV reservoirs? Curr Opin HIV AIDS 2015; 10:18–28. 10.1097/COH.0000000000000122 PMC4271317 25415421

[26] Bull L, Rayment M. HIV-indicator-condition-driven HIV testing: clinically effective but still rarely implemented. Clin Med 2016; 16:175–9. 10.7861/clinmedicine.16-2-175 PMC4952973 27037389

[27] Drumhiller K, Geter A, Elmore K, Gaul Z, Sutton MY. Perceptions of patient HIV risk by primary care providers in high-HIV prevalence areas in the Southern United States. AIDS Patient Care STDS 2020; 34:102–10. 32202928 10.1089/apc.2019.0219

[28] Crockett K, Turan B, Whitfield S, et al Patient and provider perspectives on HIV stigma in healthcare settings in underserved areas of the US South: a mixed methods study. AIDS Behav 2022; 26(Supplement 1):112–24. 34581951 10.1007/s10461-021-03470-y PMC9009188

[29] Ibitoye M, Bennett AS, Bugaghis M, et al Provider perspectives on barriers to routine HIV testing of adolescent and young adult patients in emergency department settings. Behav Med 2023; 42:204–11. 10.1080/08964289.2021.2020207 PMC9240108 34965832

[30] Simmons E, Brown M, Sly K, Ma M, Sutton M, McLellan-Lemal E. Barriers and facilitators to HIV testing in primary care among health care providers. J Natl Med Assoc 2011; 103:432–8. 21809793 10.1016/s0027-9684(15)30340-0

[31] Porter J, Boyd C, Skandari M, Laiteerapong N. Revisiting the time needed to provide adult primary care. J Gen Intern Med 2023; 38:147–55. 10.1007/s11606-022-07707-x PMC9848034 35776372

[32] Marcelin J, Tan E, Marcelin A, et al Assessment and improvement of HIV screening rates in a Midwest primary care practice using an electronic clinical decision support system: a quality improvement study. BMC Med Inform Decis Mak 2016; 16:76. 27378268 10.1186/s12911-016-0320-5 PMC4932674

[33] Miller DJ, Denizard-Thompson N, Weaver K, et al Effect of a digital health intervention on receipt of colorectal cancer screening in vulnerable patients: a randomized controlled trial. Ann Intern Med 2018; 168:550–7. 29532054 10.7326/M17-2315 PMC6033519

[34] Halket D, Dang J, Phadke A, et al Targeted electronic patient portal messaging increases hepatitis C virus screening in primary care: a randomized study. J Gen Intern Med 2022; 37:3318–24. 35230622 10.1007/s11606-022-07460-1 PMC9551157

[35] Walensky R, Reichmann W, Arbelaez C, et al Counselor- versus provider-based HIV screening in the emergency department: results from the universal screening for HIV infection in the emergency room (USHER) randomized controlled trial. Ann Emerg Med 2011; 58(Supplement 1):S126–S132.e4. 21684391 10.1016/j.annemergmed.2011.03.023 PMC3268065

[36] Salvatori C, Jeyaraju M, Schmalzle S. Sub-optimal routine HIV testing rates in an urban HIV and infectious disease clinic. Int J STD AIDS 2024; 35:254–61. 10.1177/09564624231216156 37990535

[37] Haukoos JS, Hopkins E, Bender B, et al Comparison of enhanced targeted rapid HIV screening using the Denver HIV risk score to nontargeted rapid HIV screening in the emergency department. Ann Emerg Med 2013; 61:353–61. 10.1016/j.annemergmed.2012.10.031 PMC4730951 23290527

[38] Feller DJ, Zucker J, Yin MT, Gordon P, Elhadad N. Using clinical notes and natural language processing for automated HIV risk assessment. J Acquir Immune Defic Syndr 2018; 77:160–6. 29084046 10.1097/QAI.0000000000001580 PMC5762388

[39] Krakower DS, Gruber S, Hsu K, et al Development and validation of an automated HIV prediction algorithm to identify candidates for pre-exposure prophylaxis: a modelling study. Lancet HIV 2019; 6:e696–e704. 31285182 10.1016/S2352-3018(19)30139-0 PMC7522919

[40] Burns CM, Pung L, Witt D, et al Development of a human immunodeficiency virus risk prediction model using electronic health record data from an academic health system in the southern United States. Clin Infect Dis 2023; 76:299–306. 36125084 10.1093/cid/ciac775 PMC10202432

