# Supplementary material for: Frequent Missed Opportunities for Earlier HIV Diagnosis in a Routine Opt-out Testing Environment in Atlanta
Source: Open Forum Infect Dis. 2025 Aug 26;12(8):ofaf423. doi: 10.1093/ofid/ofaf423 (PMC12378091; doi:10.1093/ofid/ofaf423)
Supplement: ofaf423_Supplementary_Data [file ofaf423_supplementary_data.zip › Supplementary_Table1.pdf]

Supplementary Table 1. Factors Predictive of Having a Missed Opportunity for HIV Screening Among Individuals Diagnosed with HIV 2012-2022

	**Missed Opportunity†**		**Univariable**		**Multivariable**	
n (%)	**No** (n=214, 30%)	**Yes** (n=499, 70%)	**OR (95% CI)**	**p-value**	**Adjusted OR (95% CI)**	**p-value**
**HIV-Related Factors**						
HIV Screened 366-730 days Before Dx	65 (30%)	68 (14%)	0.36 (0.25, 0.53)	<.0001	0.41 (0.27, 0.63)	<.0001
High HIV Incidence Zip Code Resident	171 (80%)	373 (75%)	0.74 (0.50, 1.1)	0.17	0.76 (0.49, 1.1)	0.19
CD4 <200	13 (6.1%)	63 (13%)	2.2 (1.2, 4.2)	0.014	1.7 (0.92, 3.4)	0.11
CD4 <350	29 (14%)	133 (27%)	2.3 (1.5, 3.6)	0.0002	1.8 (1.2, 2.9)	0.011
**Healthcare Utilization**						
# of Encounters 30-365 days before Dx*	3.5 (3.5)	2.2 (2.3)	0.48 (0.30, 0.75)	<.0001	0.83 (0.77, 0.89)	<.0001
# of Encounters Ever*	92 (146)	76 (121)	1.0 (0.99, 1.0)	0.13	1.0 (0.99, 1.0)	0.027
# of PC Encounters 2y≤Dx*	2.9 (9.0)	3.8 (12.9)	1.0 (0.99, 1.0)	0.31	1.0 (0.99, 1.0)	0.25
>2 visits to ED/UC 2y≤Dx	103 (48%)	142 (28%)	0.43 (0.31, 0.60)	<.0001	0.68 (0.46, 1.0)	0.054
ED Encounter without Labs 2y≤Dx	113 (53%)	332 (67%)	1.8 (1.3, 2.5)	0.0007	2.6 (1.8, 3.7)	<.0001
STI Exposure Complaint 2y≤Dx	25 (12%)	15 (3.0%)	0.23 (0.12, 0.45)	<.0001	0.43 (0.21, 0.88)	0.022
SH Related Complaint 2y ≤Dx	99 (46%)	138 (28%)	0.44 (0.32, 0.62)	<.0001	0.62 (0.42, 0.90)	0.013
SH Related Diagnosis 2y≤Dx	41 (19%)	80 (16%)	0.81 (0.53, 1.2)	0.36	1.1 (0.70, 1.9)	0.60

No SH Related Visit	107 (50%)	332 (67%)	2.0 (1.4, 2.8)	<.0001	1.4 (0.98, 2.1)	0.064
**Social Determinants of Health**						
Housing Instability	59 (28%)	133 (27%)	0.95 (0.67, 1.4)	0.87	1.0 (0.70, 1.6)	0.84
Food Insecurity	3 (1.4%)	5 (1.0%)	0.71 (0.17, 3.0)	0.94	0.44 (0.09, 2.4)	0.31
SDOH Dx	69 (32%)	164 (33%)	1.0 (0.73, 1.4)	0.94	1.1 (0.78, 1.7)	0.51
**Behavioral/Mental Health**						
Mental Health Disorder	95 (44%)	220 (44%)	0.99 (0.72, 1.4)	0.99	1.3 (0.92, 1.9)	0.13
Substance Use Disorder	103 (48%)	235 (47%)	0.96 (0.70, 1.3)	0.86	1.1 (0.76, 1.5)	0.68
Cocaine / Meth. Use	24 (11%)	43 (8.6%)	0.75 (0.44, 1.3)	0.34	0.72 (0.39, 1.3)	0.28
High Risk Sexual Behavior Noted	2 (0.93%)	2 (0.40%)	0.43 (0.06, 3.0)	0.74	0.71 (0.08, 6.4)	0.74
STI Dx	90 (42%)	174 (35%)	0.74 (0.53, 1.0)	0.082	1.1 (0.76, 1.6)	0.65
STI 2y ≤ Dx	35 (16%)	26 (5.2%)	0.28 (0.16, 0.48)	<.0001	0.47 (0.26, 0.83)	0.009
PID 2y ≤ Dx	2 (0.93%)	2 (0.40%)	0.43 (0.06, 3.0)	0.74	0.65 (0.06, 6.9)	0.71
Preventive Contraception Use	30 (14%)	36 (7.2%)	0.48 (0.29, 0.80)	0.006	0.88 (0.44, 1.8)	0.72
IV Drug Use	2 (0.93%)	8 (1.6%)	1.7 (0.36, 8.2)	0.73	2.7 (0.61, 18.7)	0.24
Emergency Contraception Use 2y ≤ Dx	7 (3.2%)	6 (1.2%)	0.36 (0.12, 1.1)	0.11	0.58 (0.16, 2.0)	0.38

Positive STI Test 2y ≤ Dx	27 (13%)	42 (8.4%)	0.64 (0.38, 1.1)	0.11	1.1 (0.60, 1.9)	0.86
Positive Gonorrhea Test 2y ≤ Dx	9 (4.2%)	13 (2.6%)	0.61 (0.26, 1.4)	0.37	1.1 (0.42, 2.9)	0.88
New Syphilis 2y ≤ Dx	9 (4.2%)	11 (2.2%)	0.51 (0.21, 1.3)	0.22	0.72 (0.27, 2.0)	0.50

* Represented as μ (σ)

†In this context, a missed opportunity is defined as an individual who was not screened for HIV in all relevant encounters 30-365 days before HIV diagnosis or screened 30 days prior. This outcome is stratified by those with all missed opportunities in the year prior to diagnosis vs. those who had no or some missed opportunities for HIV screening.

Abbreviations: SH = Sexual Health; ED = Emergency Department; UC = Urgent Care; WH = Women's Health, PC = Primary Care, STI = Sexually Transmitted Infection, SDOH = Social Determinants of Health, Meth = Methamphetamine, PID = Pelvic Inflammatory Disease, IV = Intravenous, OR = Odds Ratio, CI = Confidence Interval
