# Supplementary material for: Frequent Missed Opportunities for Earlier HIV Diagnosis in a Routine Opt-out Testing Environment in Atlanta
Source: Open Forum Infect Dis. 2025 Aug 26;12(8):ofaf423. doi: 10.1093/ofid/ofaf423 (PMC12378091; doi:10.1093/ofid/ofaf423)
Supplement: ofaf423_Supplementary_Data [file ofaf423_supplementary_data.zip › Supplementary_Table2.pdf]

*Supplementary Table 2. Encounter Characteristics: Labs, Diagnoses, and Encounter Locations on Relevant Encounters* in the Year Prior to HIV Diagnosis*

| n (%) | **Encounters** (n=1845) | **No Missed HIV Testing Opportunity** (n=610; 33%) | **Missed HIV Testing Opportunity†** (n=1235; 67%) | **Odds Ratio (95% CI)** | **p-value** |
|---|---|---|---|---|---|
| **Encounter Types** | | | | | |
| Outpatient | 762 (41%) | 213 (35%) | 549 (44%) | Ref | |
| ED/UC | 1003 (54%) | 369 (60%) | 634 (51%) | 0.67 (0.54, 0.82) | <.0001 |
| Inpatient | 80 (4.3%) | 28 (4.6%) | 52 (4.2%) | 0.72 (0.45, 1.2) | 0.19 |
| **Outpatient Departments‡** | | | | | |
| PC | 470 (62%) | 116 (55%) | 354 (65%) | Ref | |
| OB/GYN | 109 (14%) | 51 (24%) | 58 (11%) | 0.37 (0.24, 0.57) | <.0001 |
| Specialty | 183 (24%) | 46 (22%) | 137 (25%) | 0.98 (0.66, 1.5) | 0.90 |
| **Lab Tests Taken** | | | | | |
| Bacterial STI | 257 (14%) | 138 (23%) | 119 (9.6%) | 0.36 (0.28, 0.48) | <.0001 |
| Gonorrhea | 193 (11%) | 110 (18%) | 83 (6.7%) | 0.33 (0.24, 0.44) | <.0001 |
| Syphilis | 123 (6.7%) | 79 (13%) | 44 (3.6%) | 0.25 (0.17, 0.36) | <.0001 |
| Chlamydia | 196 (11%) | 110 (18%) | 86 (7.0%) | 0.34 (0.25, 0.46) | <.0001 |
| **Positive Lab Tests** | | | | | |
| Bacterial STI | 57 (3.1%) | 28 (4.6%) | 29 (2.3%) | 0.50 (0.29, 0.85) | 0.013 |
| Gonorrhea | 36 (2.0%) | 16 (2.6%) | 20 (1.6%) | 0.61 (0.31, 1.2) | 0.20 |
| Syphilis | 5 (0.3%) | 4 (0.7%) | 1 (0.1%) | 0.12 (0.01, 1.1) | 0.079 |
| Chlamydia | 32 (1.7%) | 19 (3.1%) | 13 (1.1%) | 0.33 (0.16, 0.67) | 0.003 |
| **Behavioral/Sexual Health** | | | | | |
| Non-SH Encounter | 1580 (86%) | 517 (85%) | 1063 (86%) | 1.1 (0.85, 1.5) | 0.49 |
| STD Exposure Complaint | 36 (2.0%) | 18 (3.0%) | 18 (1.5%) | 0.49 (0.25, 0.94) | 0.045 |

| | | | | | |
|---|---|---|---|---|---|
| SDOH Dx | 86 (4.7%) | 37 (6.1%) | 49 (4.0%) | 0.64 (0.41, 0.99) | 0.058 |
| High Risk Sexual Behavior | 13 (0.7%) | 7 (1.1%) | 6 (0.5%) | 0.42 (0.14, 1.3) | 0.19 |
| Mental Health Dx | 331 (18%) | 103 (17%) | 228 (18%) | 1.1 (0.86, 1.4) | 0.44 |
| Substance Use Dx | 237 (13%) | 78 (13%) | 159 (13%) | 1.0 (0.75, 1.3) | 0.99 |
| Cocaine/Meth Use | 15 (0.8%) | 7 (1.1%) | 8 (0.6%) | 0.56 (0.20, 1.6) | 0.40 |
| Preventive Contraception Prescribed | 20 (1.1%) | 9 (1.5%) | 11 (0.9%) | 0.60 (0.25, 1.5) | 0.37 |
| Emergency Contraception Prescribed | 2 (0.1%) | 2 (0.3%) | 0 (0%) | 0.10 (0.001, 1.2) | 0.072 |

*Relevant encounters are 30-365 days before HIV diagnosis that were in primary care, ED, UC, or inpatient.

†In this context, a missed HIV testing opportunity is defined as an encounter 30-365 days before HIV diagnosis in which they were not screened within 6 months or not screened upon an encounter in which there was a sexual health related chief complaint or diagnosis.

‡ PC, OB/GYN, and Specialty are departments from outpatient encounters. Therefore, their column % only considers outpatient encounters.

Abbreviations: STI = Sexually Transmitted Disease (encompasses Gonorrhea, Syphilis, or Chlamydia), STD = Sexually Transmitted Disease, SH = Sexual Health, Meth = Methamphetamines, SDOH = Social Determinants of Health, Dx = Diagnosis ED = Emergency Department, UC = Urgent Care, PC = Primary Care, OB/GYN = Obstetrician and Gynecologist, Specialty = Specialty Clinics such as Dermatology, Cardiologists, etc.
